# Aging and Vision Loss: Biopsychosocial Perspectives

**DOI:** 10.1093/geroni/igaf122.1738

**Published:** 2025-12-31

**Authors:** Silvia Sörensen, Su-I Hou

## Abstract

The risk of severe vision problems increases significantly with age. Older adults with vision impairment are at increased risk for falls and hospitalization; they find it more difficult to take their medications properly, and they are more likely to be socially isolated, placing them at greater risk for physical health problems. In addition, low vision reduces individuals’ ability to engage in valued and enjoyable activities, thus increasing their vulnerability to depression and anxiety. Vision rehabilitation that assesses the patients’ current abilities and goals and provides rehabilitative training is available to those of working age with legal blindness, but access is more limited to adults 65+ and to those with low vision, but who are not legally blind. Furthermore, emotional adjustment to vision loss is less consistently addressed in rehabilitation contexts, placing the vision-impaired older adult at risk for unrecognized mental health problems. In this interdisciplinary symposium, Donaher will describe the changes in the eye that occur with aging. Sörensen will describe the psychological effects of vision loss in late life, interventions that can ameliorate these effects, and cultural adaptation of such interventions to a highly underserved population, Latino older adults. Chang investigates the potential factors contributing to loneliness among vision impaired older adults. Cai and Powers will report changes among treatment and control groups in dimensions of psychological well-being in a clinical trial of a psychosocial intervention. The discussant will reflect upon the implications of these findings for clinical care and future research needs.

